# Abundance of Entomopathogenic Fungi in Leaf Litter and Soil Layers in Forested Habitats in Poland

**DOI:** 10.3390/insects12020134

**Published:** 2021-02-05

**Authors:** Anna Majchrowska-Safaryan, Cezary Tkaczuk

**Affiliations:** Faculty of Agrobioengineering and Animal Husbandry, Institute of Agriculture and Horticulture, Siedlce University of Natural Sciences and Humanities, 08-110 Siedlce, Poland; cezary.tkaczuk@uph.edu.pl

**Keywords:** insect-pathogenic fungi, Hypocreales, *Beauveria*, colony-forming units, habitat preference

## Abstract

**Simple Summary:**

The soil environment is an important reservoir for a wide variety of entomopathogenic fungi that can suppress insect populations, including agricultural and forestry pests. This research aims to investigate the species composition and density of entomopathogenic fungi (EPF) in the leaf litter and at different soil depths in different types of forests during different seasons (spring, autumn). The current study describes the density of four different genera of entomopathogenic fungi in forest soils and leaf litter. The densities of EPF were usually higher in leaf litter than soil, and *Beauveria* spp. were the most prevalent fungi among leaf litter and soil samples compared to other entomopathogenic fungi. This research will give new insights into our understanding of EPF diversity and composition in forests.

**Abstract:**

This study aims to determine the species composition and density of colony-forming units (CFU) of entomopathogenic fungi (EPF) in leaf litter at different depths of the top layer of forest soils depending on the type of forest (coniferous, deciduous and mixed forest), and the date of sampling (spring, autumn). In each type of forest, leaf litter and soil were collected using a soil stick from four depths of soil: 0–5, 5–10, 10–15 and 15–20 cm. Entomopathogenic fungi were isolated by a soil or litter dilution plating method on a selective medium. Four fungal genera were found: *Beauveria* spp., *Cordyceps* spp., *Metarhizium* spp., and *Lecanicillium* spp. The density of EPF was usually higher in leaf litter than in the layers of soil below, and the most frequently isolated species from both environments were *Beauveria* spp. among soil samples from all forest types; *Beauveria* spp. were most abundant in the top layer (0–5 cm), and their density of CFUs gradually decreased deeper into the soil profile.

## 1. Introduction

Forests cover over 29% of Poland’s territory [1]. The stands are dominated mainly by conifers, including *Pinus sylvestris* L., which covers about 60% of forest areas [2]. The predominance of coniferous stands results in favourable conditions for the development and gradation of pests in single-species and equal-age forests growing in poor and degraded habitats [3]. It is believed that over 65% of Poland’s biological resources are concentrated in forest ecosystems. One of the characteristic features of soil is its biological activity, shaped by a number of microorganisms, including entomopathogenic fungi (EPF) [4,5].

The soil environment is an important reservoir for a wide variety of EPF that can make an important contribution to the control of insect populations, including agricultural and forestry pests [6]. The occurrence and distribution of EPF in soils of various environments have been the subject of numerous studies in many countries [4,7,8,9,10,11]. Many species of Hypocrealean (Ascomycota) fungi live in the soil for most of their life cycle. Among these fungi, *Beauveria* spp., *Metarhizium* spp., *Cordyceps* spp. and *Lecanicillium* spp. are common genera found in agricultural and forest soils and have the greatest potential for biological control [12,13]. These fungi form a specialised group of natural enemies that can infect host populations on a large scale and lead to epizootics [14,15]. Entomopathogenic fungi inhabiting the soil environment are important bioregulators not only of typical soil pests but also of a large group of pests of agricultural and forestry crops that descend into the soil for periodic diapause, pupation or wintering. It is estimated that about 90% of herbivorous pests spend at least part of their development cycle in the soil [16]. Arthropod hosts are killed by EPF and, with fungal outgrowth, provide inocula that can spread to other susceptible hosts in the soil environment, e.g., horizontal transmission. According to Bałazy [17], the soils of seminatural environments constitute an important reservoir of EPF populations in the natural landscape, from which they can spread into the habitats of cultivated fields and meadows. Entomopathogenic fungi provide an invaluable service of suppressing pest populations and preventing pest outbreaks in forest habitats [14,18,19,20].

Identifying native strains of EPF and studying their population densities (CFUs) and patterns of occurrence in forest leaf litter and the soil environment may provide insights into the biodiversity of naturally occurring fungi and may expand the pool of potential species used in biological control [21].

Therefore, this study aims to determine the species composition and intensity of EPF occurrence in leaf litter and soils in selected forested habitats in Poland.

## 2. Materials and Methods

### 2.1. Research Location

The material for the study consisted of leaf litter and forest soils collected between 2015 and 2017 from the areas of the State Forests, Siedlce Forest District, Masovian Voivodeship, Poland, in the villages of Golice (geographical coordinates 52°12′15″ N and 22°20′32″ E) and Chodów (geographical coordinates 52°12′42″ N and 22°14′05″ E), located approximately 10 km from each other (Figure S1). In the years of sampling for research, different weather conditions prevailed (Table S1).

Samples for research were collected at two localities in time: autumn (I-2015, III-2016 October) and spring (II-2016, IV-2017 May). In each of them, in the forest complex, three types of forest habitats were selected (coniferous, deciduous and mixed forests), with an area of about 10 hectares each. The first type of forest was represented by coniferous pine forests, the second by deciduous forests with a predominance of oak, and the third was a mixed forest with a predominance of deciduous trees. The forests in Golice and Chodów were about 50 and 40 years old, respectively. In each type of forest, leaf litter and soil were collected using a soil stick (Ø 30 mm) from four layers: 0–5, 5–10, 10–15 and 15–20 cm. At each site, leaf litter and soil were collected from 15 randomly selected sites, including a minimum distance of 10 m between them. The soil stick was cleaned with 70% ethanol between sampling points. A mixed sample was prepared from the samples collected in this way (in total, 200 g of leaf litter and 300 g of soil from each layer was taken from each forest type and location) and stored in plastic bags at the temperature of 0–4° C.

### 2.2. Soil Analysis

In the collected material, selected chemical properties were determined in the laboratory of the Regional Chemical and Agricultural Station in Warsaw in accordance with the applicable standards and procedures: pH of soil and leaf litter using the potentiometric method in 1 mol KCl; organic matter content in the leaf litter—by weighted method, after drying at 105 °C and burning the analysed material in a muffle furnace at 550 °C (Table S2).

### 2.3. Fungi Isolation

EPF from leaf litter and individual layers of soil to a depth of 20 cm were isolated by sowing on a selective culture medium developed by Strasser et al. [22]. This is a commonly used method for the isolation of entomopathogenic fungi from soil [9,23,24,25,26] and leaf litter environments [27] and is particularly useful when quantification is necessary. The selective medium was composed of 20 g of glucose (Biomus, Białystok, Poland), 10 g of peptone (Becton, Dickinson and Company, Le Pont de Claix, France) and 18 g of agar (Sigma, St. Louis, USA), which were dissolved in 1 L of deionised water, and then sterilised in an autoclave at 120 °C for 20 min. The following selective components were added to the medium after it cooled to 50 °C: 0.6 g of streptomycin (Serva, Heidelberg, Germany), 0.05 g of tetracycline (Sigma, St. Louis, MO, USA), 0.05 g of cycloheximide (Sigma, St. Louis, MO, USA), and 0.1 g of dodine (Arysta LifeScience, Seraing, Belgium). For each mixed sample of leaf litter or layer of soil, 2 g of soil or leaf litter were mixed with 18 mL of 0.05% nonsterilised Triton-X (Sigma, St. Louis, MO, USA). Using an automatic pipette, soil solutions prepared in the amount of 0.1 mL were placed on the surface of the selective substrate and spread with the use of a glass spatula. Petri dishes (90 mm, Noex, Komorniki, Poland) were placed in incubators at 22 °C, and, after 8–10 days, the colonies of fungi were counted. We used three replicates of selective medium for each sample. The results are expressed as the number of CFUs of each genera of entomopathogenic fungi in 1 g of dry leaf litter or soil.

The in-vitro fungal cultures were microscopically identified according to the morphology of the microstructures [28,29,30,31]. Characterisation of fungal isolates was made by the determination of conidial size and shape, conidiogenous cell, and colony morphology. Given that only morphological methods were applied during the identification of fungi, they were described to the rank of genus, because, as demonstrated by the latest phylogenetic studies based on DNA sequencing [29,32,33,34], there are numerous fungus species within the genus of *Beauveria*, *Cordyceps*, *Metarhizium* and *Lecanicillium* that are almost impossible to distinguish from each other without the application of molecular methods.

### 2.4. Statistical Analysis

The obtained results were statistically processed using the Statistica program v. 13.3 (TIBCO Software Inc, Palo Alto, CA, USA). The first stage of the analysis was to check with the chi-square λ^2^ test—whether the distribution of the tested trait (number of CFUs) in the sample followed a normal distribution. Since the data did not have a normal distribution, the transformation y = log (x + 0.5) was applied. Then, on the transformed data, one-way ANOVA analysis was performed for each factor separately according to the following model:

y_ij_ = m + a_i_ + e_ij_;

where m is the population average; y_ij_ is the value of the examined trait (number of CFUs); a_i_ is the effect of the i-th level of factor A (sampling date, type of forest, sampling depth); e_ij_ is the random error.

When the factor’s effect was significant, the Tukey test was used at *p* ≤ 0.05 to compare the means (posthoc analysis).

## 3. Results

In the samples of leaf litter and soil, the presence of four genera of EPF was found: *Beauveria* spp., *Cordyceps* spp., *Metarhizium* spp., and *Lecanicillium* spp. (Table 1 and Table 2).

**Figure 1:**
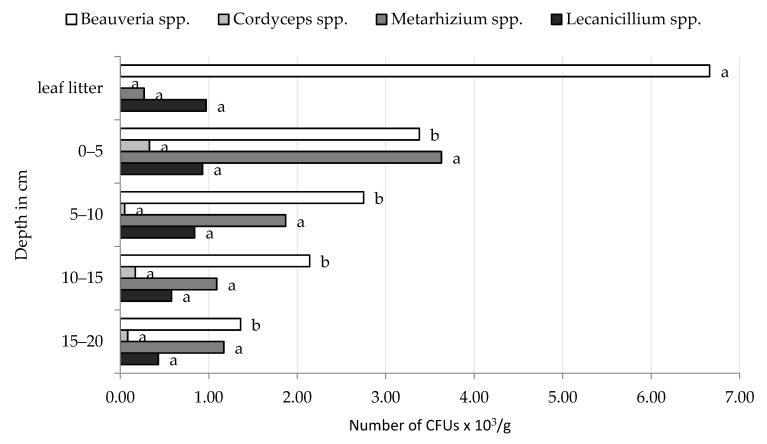
Average density of CFUs of entomopathogenic fungi in the leaf litter and individual layers of forest soils collected in Golice. ^ab^ Statistically significant differences between the concentration of CFUs of entomopathogenic fungi depending on the soil depth.

**Figure 2:**
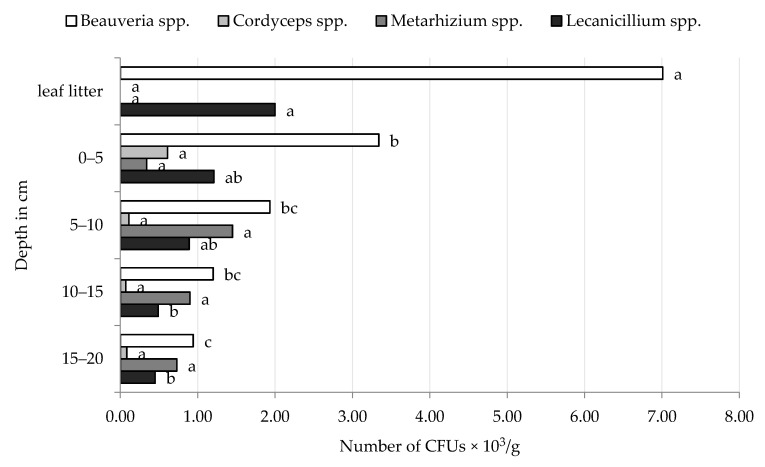
Average density of CFUs of entomopathogenic fungi in the leaf litter and individual layers of forest soils collected in Chodów. ^abc^ Statistically significant differences between the concentration of CFUs of entomopathogenic fungi depending on the soil depth.

The density of EPF in the forest leaf litter varied depending on the sampling date and the type of forest, but the dominant genus was *Beauveria*. In both Golice and Chodów forests, *Beauveria* spp. and *Lecanicillium* spp. were isolated from samples on all sampling dates (Table 1 and Table 2). The greatest density of *Beauveria* spp. in leaf litter occurred in the spring of 2017. We observed an increasing trend of higher densities of *Beauveria* spp. in leaf litter samples over subsequent sampling dates. The highest density of CFUs of *Beauveria* spp. fungi, regardless of the date of the study, was observed in the leaf litter collected from the Golice locality, from the mixed forest (10.0 × 10^3^ CFUs/g), while in the village of Chodów, from the deciduous forest, these fungi produced 13.2 × 10^3^ CFUs per g leaf litter. *Lecanicillium* spp. were recovered from leaf litter samples in Golice forestes on all sampling dates, with mean densities ranging from 0.3 to 2.2 × 10^3^ CFU/g. We found greater densities of *Lecanicillium* spp. in the leaf litter in Chodów than the leaf litter in Golice (Figure 1 and Figure 2). The fungi from *Metarhizium* genus were also found in the leaf litter in Golice; the fungi formed CFUs in the first (autumn 2015) study period, in deciduous leaf litter, and in the second study period (spring 2016), in coniferous leaf litter. In deciduous leaf litter collected in spring 2017 from Chodów, apart from *Beauveria* spp. and *Lecanicillium* spp., the presence of two more genera of EPF was noted, namely, *Cordyceps* spp. and *Metarhizium* spp. Both species produced of 0.2 × 10^3^ CFUs per g leaf litter.

The current study showed that the mean densities of EPF varied by sampling date, soil depth, and forest type (Table 1 and Table 2).

The research showed that the *Beauveria* spp. were most abundant in the top layer of the soil (0–5 cm), and densities of these fungi gradually decreased deeper into the soil profile (Table 1 and Table 2). When analysing the number of CFUs of these fungi, at a soil layer of 0–10 cm, *Beauveria* spp. in the first two study dates (autumn 2015 and spring 2016) formed at least twice as many CFUs than in the soil layer below (10–20 cm). In the soils collected in autumn 2016, this difference was several times higher. The largest number of *Beauveria* spp. was found in a soil layer of 0–5 cm in autumn 2016 from a deciduous forest located in Chodów; the density was 9.0 × 10^3^ CFUs/g. Regardless of the type of forest and locality, the smallest number of CFUs of *Beauveria* spp. was formed in autumn 2015 (Table 1 and Table 2; Figure 1 and Figure 2).

Second to *Beauveria* spp., we frequently isolated *Lecanicillium* spp. from soil samples in both localities. The research showed that the number of CFUs of these fungi in each layer of the studied levels showed a tendency to decrease deeper into the soil profile, with few deviations. The fungi *Lecanicillium* spp. in the soil layer, lying 0–5 and 5–10 cm under the leaf litter, formed, on average, twice as many CFUs than the fungi in the lower layers (Table 1 and Table 2).

The presence of the fungi from the genus *Cordyceps* was also noted in individual samples of the investigated forest soils. It is worth noting that the presence of CFUs of these fungi was not found in any of the examined soil layers from the pine forest at all four test dates in both localities.

The occurrence and density of *Metarhizium* fungi varied by forest type and locality. The greatest number of CFUs was formed by these fungi in the soil collected from the coniferous forest in Golice in two study periods (autumn 2015 and spring 2016) in the 0–5 cm layer, creating 28.8 × 10^3^ and 11.5 × 10^3^ CFUs/g, respectively. In the layers below, deeper into the soil profile, a tendency of a gradual reduction in the concentration of CFUs of this species was observed (Table 1). In contrast, *Metarhizium* spp. were most abundant in deeper layers of soil in deciduous forests in Chodów compared to mean CFUs in the top layer (0–5 cm; Table 2, Figure 2).

Among the EPF that were isolated from forested habitats, *Beauveria* spp. and *Lecanicillium* spp. were most abundant in leaf litter; their density in soil gradually decreased in deeper soil depths (Figure 1 and Figure 2).

**Figure 3:**
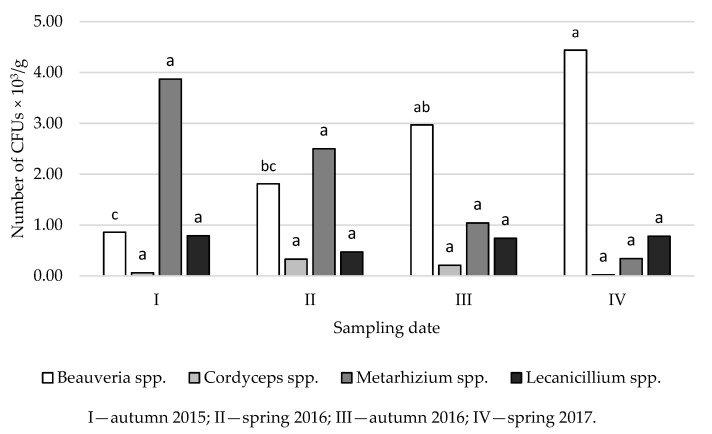
Average density of CFUs of entomopathogenic fungi in forest soils collected in Golice depending on the date of sampling for research. ^abc^ Statistically significant differences between the concentration of CFUs of entomopathogenic fungi in the studied soils and the time of the study.

**Figure 4:**
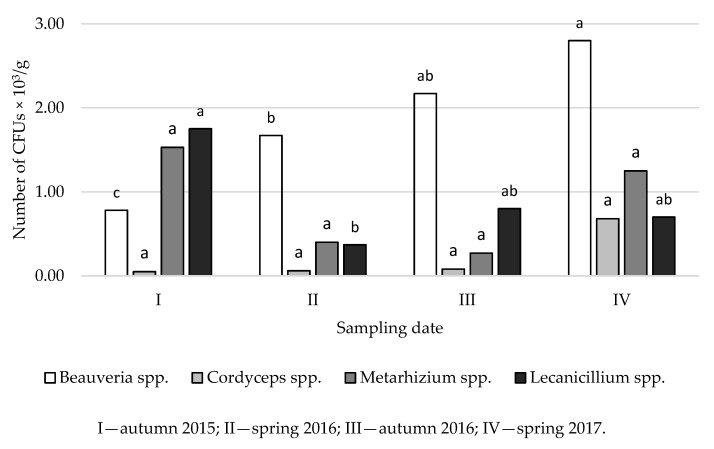
Average density of CFUs of entomopathogenic fungi in forest soils collected in Chodów, depending on the date of sampling for research. ^abc^ Statistically significant differences between the concentration of CFUs of entomopathogenic fungi in the studied soils and the time of the study.

The current study shows that the abundance of EPF varies depending on the time of the study (Figure 3 and Figure 4). The highest densities of *Beauveria* spp. and *Lecanicillium* spp. were found in Golice in the spring of 2017, and the greatest abundance of *Cordyceps* spp. was observed in spring 2016. *Metarhizium* spp. were most abundant in autumn 2015 compared to other sampling dates (Figure 3).

In soil samples collected from Chodów, the greatest densities of *Beauveria* spp. and *Cordyceps* spp. occurred in spring 2017, whereas the greatest densities of *Metarhizium* spp. and *Lecanicillium* spp. were observed in autumn 2015 (Figure 4). The type of environment (coniferous, deciduous and mixed forest), both in Golice and Chodów, did not significantly affect the density of EPF. However, the main effects of sampling date and sample/depth were significant (Figure 1, Figure 2, Figure 3 and Figure 4). As a result of the analysis of variance, in both locations, the sampling depth and the sampling time significantly affect the number of CFUs of *Beauveria* spp.; in Chodów, this included *Lecanicillium* spp. These differences were not statistically significant regarding *Cordyceps* spp. and *Metarhizium* spp.

## 4. Discussion

The soil environment provides a suitable habitat for insect-pathogenic fungi and other microorganisms since it is protected from UV radiation and buffered against extreme biotic influences [6]. An understanding of the parameters that determine the diversity and distribution of entomopathogenic fungal species in soil would help to identify those species best suited to a particular environment and improve biological control efficacy.

In our research on leaf litter and soil collected from different types of forests in Golice and Chodów, the presence of four genera of EPF was found: *Beauveria* spp., *Cordyceps* spp., *Metarhizium* spp., and *Lecanicillium* spp. The density of EPF in leaf litter and soil was explained by the time of sampling and the depth of soil samples. Moreover, it was found that the density of EPF was usually higher in leaf litter than in the layers of soil below, and the most frequently isolated fungus from both environments was *Beauveria* spp. The research carried out as part of this study showed that *Beauveria* spp. often formed several times more CFUs in leaf litter than in the underlying layers of forest soil. The obtained results confirm the research conducted by Tkaczuk et al. [27], who, carrying out investigations on the density of EPF in three mixed forest habitats in Poland, separately for leaf litter and the soil layer below, found that the fungus *B. bassiana* dominated in both environments but formed more CFUs in the leaf litter layer than in the soil. Bajan et al. [35], studying leaf litter and soil originating from pine forests in Poland, found that the fungus species that, to the greatest extent, causes the death of the larvae of the trap insect was *B. bassiana*. Tkaczuk [4] and Karg and Bałazy [36] found that forest environments favour the persistence of EPF, and, compared to agroecosystems, forests are more than twice as rich in EPF abundance, with high infectious potential.

The dominance of *B. bassiana* in soils collected from various types of forests is also confirmed by studies conducted in Denmark [37], Finland [7], Poland [27,35,38,39], Japan [40], Italy [41,42] Spain [43,44], Austria [45], Mexico [46], Brasil [47] and Portugal [48]. Niemczyk et al. [21], examining the occurrence of *Beauveria* spp. in forest soils in Poland, found that this fungus was isolated from a majority of soil samples. As mentioned earlier in the methodology, our research only used morphological methods to determine fungi; this is currently insufficient to differentiate species within the *Beauveria,* genus [29,32,33], and it is certain that within the isolates of fungi of the genus *Beauveria* isolated by us on a selective medium, there are several species other than *B. bassiana*. So far, five *Beauveria* species, including *B. bassiana*, *B. brongniartii*, *B. caledonica*, *B. varroae* and *B. pseudobassiana,* have been documented in Europe [49].

Leaf litter and the soil layer under it are the main reservoirs of EPF in forest environments [4,25,35,39,50,51,52]. Entomopathogenic fungi infecting forest insects can easily survive in leaf litter and on the soil surface, where they have a chance to come into contact, all year round, with hosts that constantly live in the forest floor environment or use them only as a place for pupation or wintering. The periodic or continuous development of EPF in the surface layers of forest soil and leaf litter is, in many cases, a condition for the survival of the species and the spread of pathogens [14,18,27]. *B. bassiana*, in its development cycle, seems to use the strategy, which is referred to by Ewald [53,54] as “sit and wait”, which means that the development of the population and the survival of this species in soil or leaf litter depend mainly on repeated host infections over time, and the factors that limit the host population appear to have a major influence on the survival of *B. bassiana* in the soil environment. The studies of Daousta and Pereira [55] and Steenberg [37] indicate that the continued presence of arthropods in the soil, which are potential *B. bassiana* hosts, has a significant impact on the survival of this species.

*B. bassiana* is a fungus often mentioned by many authors as a pathogen of insects temporarily staying or hibernating in the upper layers of forest soil and leaf litter. This species accounted for more than 85% of the total mortality of *Diprion pini* L. [39] larvae wintering in cocoons and was the most frequently isolated entomopathogen from forest insects in Finland [7]. It should be remembered that leaf litter is an environment very rich in organic matter (Table S2); therefore, one of the concepts explaining the dominance of *Beauveria* spp. in the layers of litter, rich in organic matter, is the ability of this species to develop in the saprophagous phase [4,7,8].

In the soil environment, EPF show a varying intensity of occurrence depending on the season [25,56]; the factors determining their occurrence include temperature and moisture levels [25,56,57,58]. Bruck and Lewis [59] showed the significant effect of precipitation intensity and, thus, humidity levels on the growth and sporulation of EPF. In the current study, it was observed that the fungi *Beauveria* spp. produced the fewest CFUs regardless of the type of forest and the locality in autumn 2015. A factor that influenced such results may have been the relatively dry summer and autumn recorded in 2015, when total rainfall was 7.5 mm in August and 22.8 mm in October, respectively (Table S1).

The second genus that, apart from *Beauveria* spp., was the most numerous was *Lecanicillium* spp. This is the first study to uncover such a high abundance of this fungus in the leaf litter and forest soils in Poland. Entomopathogenic fungi species belonging to the genus *Lecanicillium* (formerly *Verticillium lecanii*) have a global distribution, occur on a diverse range of insect species, and have potential for their development as effective biological control agents against a number of plant diseases, insect pests and plant-parasitic nematodes [60]. This entomopathogenic fungus was previously isolated from forest soils in Poland [4,25], China [61], Mexico [62] and India [52].

The current study shows that *Metarhizium* spp. and *Cordyceps* spp. occupy forest soils; however, the frequency of their isolation was much lower than that of *Beauveria* spp. and *Lecanicillium* spp. *M. anisopliae* is generally more resistant to agricultural disturbances, and numerous studies have reported that it is significantly more prevalent in cultivated areas than in natural habitats [8,56,63,64]. The current study shows that, especially in autumn, in the case of soils from the deciduous and mixed forests in Chodów, the CFU density of the fungi *Metarhizium* spp. and *Cordyceps* spp. is sometimes higher in deeper soil layers than in shallow ones. This may be due to the increased activity of insects in the soil environment of forests during summer and autumn and, thus, the transfer of spores and other forms of fungal propagation on their bodies deeper into the soil profile [65,66]. In addition, insects that go down to the soil in autumn for the winter diapause become infected and die as a result of fungal infection in the soil at different depths, contributing significantly to its enrichment with infectious material. According to Sosnowska et al. [67], such mycoses of various insects overwintering in the soil of deciduous and mixed forest habitats of the Białowieża Primeval Forest (Poland) are mainly caused by fungi of the genus *Cordyceps*, and epizootics caused by these fungi are often observed in autumn.

Popowska-Nowak et al. [25], who conducted research in various regions of Poland, showed that *M. anisopliae* and *I. fumosorosea* (*Cordyceps fumosorosea*) were the most frequently isolated species of entomopathogenic fungi from the soils of several-year-old forest nurseries, especially in the springtime, which, according to the authors, was associated with the higher humidity of the soil environment. Different research results by other authors may result from the fact that the species composition and the intensity of the occurrence of EPF in the soil are influenced by many factors, such as the content of organic matter, pH, soil type and method of its cultivation [7,8,9,21,47,48,68,69], as well as microclimatic conditions (temperature, humidity) and potential host density [36,68]. It could also be related to the methods of quantifying EPF abundance. Two methods are generally used to detect EPF in soil: (1) Bait methods use *Galleria mellonella* L. (Lepidoptera: Pyralidae) or other insects [46,70] and are relatively simple and sensitive but provide rather semiquantitative data [71,72,73]; (2) plating methods using various selective media [22,40,74,75,76] that are particularly useful when quantification is necessary or when the input material is not soil (e.g., air and some plant parts). Bueno-Pallero et al. [48] observed different patterns of EPF occurrence by using different isolation methods, particularly between insect baits and selective medium methodology. The use of selective media resulted in higher recoveries of EPF than either soil-baiting method. Since the aim of our research was to accurately quantify the density of fungal inoculum in individual samples of soil and leaf litter, we applied the selective medium developed by Strasser et al. [22].

The current study shows that the number of CFUs of EPF in soil from various types of forests is, in most cases, higher in the top layer of soil (0–5 and 5–10 cm), located directly under the leaf litter, and gradually decreased deeper into the soil profile. This is confirmed by the research of Tkaczuk [4], who stated that, especially in spring, in the case of forest and meadow habitats and arable fields, the highest CFUs of EPF are located in the surface layers of the soil profile (0–5 cm) and, relatively lower, at deeper depths (15–20 cm). A similar pattern was observed in autumn regarding permanent habitats such as meadows and forests. The above structure of the distribution of EPF in the soil profile in the case of meadow and forest habitats, which was also confirmed in our research, results from the stability of these environments, which, unlike arable fields, are not subjected to disturbances from farming practices.

According to Tkaczuk [4], in the soil collected in autumn from the deeper layers (15–20 cm) of cultivated fields, more CFUs were found in 1 g of soil than in the surface layer (0–5 cm), which is certainly the result of soil mixing and, thus, displacement of the infection material from the soil layers to the deepest level as a result of autumn cultivation, especially ploughing. According to Dighton et al. [77], the movement of soil as a result of tillage operations (ploughing, use of a disc harrow) may contribute to increased dispersion of soil microorganisms in the arable soil layer (20–30 cm) as well as horizontal dispersion into its deeper levels.

## 5. Conclusions

Knowledge of the composition and distribution of native entomopathogenic fungal species is essential to evaluate the potential of biological control in a given ecosystem. As part of this study, a comparative assessment of the occurrence of EPF was carried out separately for the environment of leaf litter and the forest soil directly below it in three different types of forest for the first time on a large scale. *Beauveria* spp., *Cordyceps* spp., *Metarhizium* spp., and *Lecanicillium* spp. were found in the soil and leaf litter of the investigated forests. The conducted research showed that the density of CFUs of EPF was usually higher in leaf litter than in the layers of soil below, and the most frequently isolated fungi from both environments were *Beauveria* spp. The second fungal genus that, apart from *Beauveria* spp., was the most abundant in Polish forests was *Lecanicillium* spp. The current research will give new insight into the understanding of EPF distribution and persistence in the forest underground environment and their biodiversity conservation.

## Figures and Tables

**Table 1:** Mean densities of entomopathogenic fungi (CFUs × 10^3^/g soil or leaf litter) from the Golice locality; lowercase letters represent statistically significant differences between CFUs among the sampling depths (leaf litter vs. other layers) at each sampling date and forest type.

Type	Depth (cm)	Entomopathogenic Fungi															
		*Beauveria* spp.				*Cordyceps* spp.				*Metarhizium* spp.				*Lecanicillium* spp.			
		Sample Collection Date															
		I	II	III	IV	I	II	III	IV	I	II	III	IV	I	II	III	IV
Coniferous	*L. l	3.5 a	6.7 a	7.7 a	8.2 a	0	0	0	0	0 d	0.2 d	0 c	0 c	2.2 a	0.7 a	0.8 a	1.2 a
	0–5	0.7 b	5.3 a	5.2 b	8.8 a	0	0	0	0	28.8 a	11.5 a	0.2 c	2.3 a	0 b	0.6 a	0.8 a	0.3 b
	5–10	0.5 b	2.3 b	4.2 b	8.5 a	0	0	0	0	9.2 b	8.7 b	4.0 b	0.3 bc	0 b	0.5 ab	0.5 ab	0.2 b
	10–15	0.3 b	1.8 b	2.5 c	6.7 b	0	0	0	0	4.3 c	7.1 b	1.0 c	0.5 b	0 b	0.2 b	0.2 b	0.8 ab
	15–20	0.2 b	0.5 c	0.8 d	6.8 b	0	0	0	0	3.5 c	2.8 c	7.3 a	0.5 b	0 b	0.2 b	0.2 b	0.3 b
Deciduous	L. l	2.2 b	4.7 a	9.5 a	9.8 a	0	0 c	0	0	3.0 a	0	0	0	0.3 c	1.0	0.5	0.8
	0–5	3.0 a	4.3 a	3.5 b	3.7 b	0.2	2.3 a	0.7	0	0.3 b	0	0	0.5	2.1 a	0.7	1.3	1.0
	5–10	1.7 ab	2.3 b	3.3 b	2.8 b	0	0.2 c	0.2	0	0.2 b	0	0	0	1.8 ab	0.8	0.8	0.8
	10–15	0.3 c	1.5 bc	3.2 b	3.3 b	0	1.5 b	0.3	0	0.2 b	0	0	0	1.3 b	0.5	0.8	0.8
	15–20	0.2 c	0.8 c	2.2 b	1.5 c	0.3	0 c	0.7	0	0 b	0	0	0	0 c	0.7	0.8	0.5
Mixed	L. l	2.3 a	7.5 a	9.8 a	10.0 a	0	0	0	0	0	0	0	0	0.7 ab	0.8 a	1.5	1.2
	0–5	1.5 b	2.0 b	4.3 b	3.7 b	0.2	0	0.5	0	0	0	0	0	1.6 a	0.7 a	0.8	1.3
	5–10	0.6 c	0.7 c	3.2 b	3.0 bc	0	0	0	0.2	0	0	0	0	1.4 a	0.5 ab	1.3	1.5
	10–15	1.0 b	0.3 cd	2.3 c	2.5 c	0	0	0.2	0	0	0	0	0	0.8 ab	0.2 bc	0.7	0.7
	15–20	0.3 c	0 d	1.0 c	2.0 c	0	0	0	0	0	0	0	0	0.5 b	0 c	0.7	1.2

I—autumn 2015; II—spring 2016; III—autumn 2016; IV—spring 2017; *L. l—leaf litter.

**Table 2:** Mean densities of entomopathogenic fungi (CFUs × 10^3^/g soil or leaf litter) in the Chodów locality; lowercase letters represent statistically significant differences between CFUs among the sampling depths (leaf litter vs. other layers) at each sampling date and forest type.

Type	Depth (cm)	Entomopathogenic Fungi															
		*Beauveria* spp.				*Cordyceps* spp.				*Metarhizium* spp.				*Lecanicillium* spp.			
		Sample Collection Date															
		I	II	III	IV	I	II	III	IV	I	II	III	IV	I	II	III	IV
Coniferous	*L. l	7.7 a	2.7 b	8.0 a	11.8 a	0	0	0	0	0	0	0	0	4.8 a	0.3	2.0 a	1.3
	0–5	0.5 b	5.5 a	3.2 b	5.0 b	0	0	0	0	0	0	0	0	0 b	0.8	1.5 b	0.8
	5–10	0.3 b	3.2 ab	1.3 c	4.6 bc	0	0	0	0	0	0	0	0	0 b	0.5	0.7 c	0.8
	10–15	0.2 b	2.3 b	0.5 d	2.3 bc	0	0	0	0	0	0	0	0	0 b	0.3	0.3 c	1.2
	15–20	0.2 b	0.3 c	0.3 d	2.5 bc	0	0	0	0	0	0	0	0	0 b	0.2	0.2 c	1.0
Deciduous	L. l	4.3 a	2.2 b	9.5 a	13.2 a	0	0	0	0.2 b	0 d	0	0 b	0.2 c	1.2 bc	0 c	2.2 a	1.2 a
	0–5	0.7 b	3.7 a	9.0 a	2.0 b	0.2	0.3	0	6.3 a	2.8 c	0	1.0 ab	0 c	4.3 a	0 c	2.2 a	0 b
	5–10	0.5 b	1.7 bc	4.0 b	1.0 b	0	0.2	1.0	0.2 b	4.3 b	0	1.5 a	11.8 a	1.8 bc	0.5 b	1.7 ab	1.7 a
	10–15	0.5 b	0.7 c	1.7 c	1.5 b	0	0.2	0	0.3 b	6.0 a	0	0.5 ab	0 c	2.0 b	0.3 b	0.8 b	0 b
	15–20	0.2 b	0.5 c	1.3 c	1.8 b	0	0	0	0.7 b	5.3 ab	0	0.2 b	3.2 b	1.0 c	1.0 a	0.7 b	0 b
Mixed	L. l	8.0 a	2.5 a	5.5 a	8.8 a	0	0	0	0	0	0 b	0	0	5.6 a	0.5 a	3.2 a	1.7 a
	0–5	2.5 b	1.5 b	2.5 b	4.0 b	0	0	0	0.5	0	0.3 b	0	0	3.2 b	0.3 a	0.5 b	1.0 ab
	5–10	1.2 c	0.7 bc	1.2 bc	3.5 b	0	0	0	0	0	0.3 b	0	0	1.0 c	0.5 a	0.2 b	1.3 ab
	10–15	1.5 c	0 c	0.5 c	2.7 b	0.2	0	0	0.2	0	4.3 a	0	0	0 d	0 b	0.5 b	0.5 c
	15–20	1.0 c	0 c	0.5 c	2.7 b	0.2	0	0	0	0	0 b	0	0	0.8 c	0 b	0.3 b	0.2 c

I—autumn 2015; II—spring 2016; III—autumn 2016; IV—spring 2017; *L. l—leaf litter.

## Data Availability

The data presented in this study are available in the article and Supplementary Materials provided.

## Supplementary Materials

The following are available online at https://www.mdpi.com/2075-4450/12/2/134/s1, Figure S1: Location of sampling for testing; Table S1: Average values of temperature and precipitation in 2015–2017 (Siedlce district); Table S2: The pH value and the content of organic matter in the forest litter and soil samples (average value from the 0–20 cm layer) of the studied forest types.
