# Supplementary material for: Abundance of Entomopathogenic Fungi in Leaf Litter and Soil Layers in Forested Habitats in Poland
Source: Insects. 2021 Feb 5;12(2):134. doi: 10.3390/insects12020134 (PMC7915602; doi:10.3390/insects12020134)
Supplement: Supplementary file 1 [file insects-12-00134-s001.pdf]

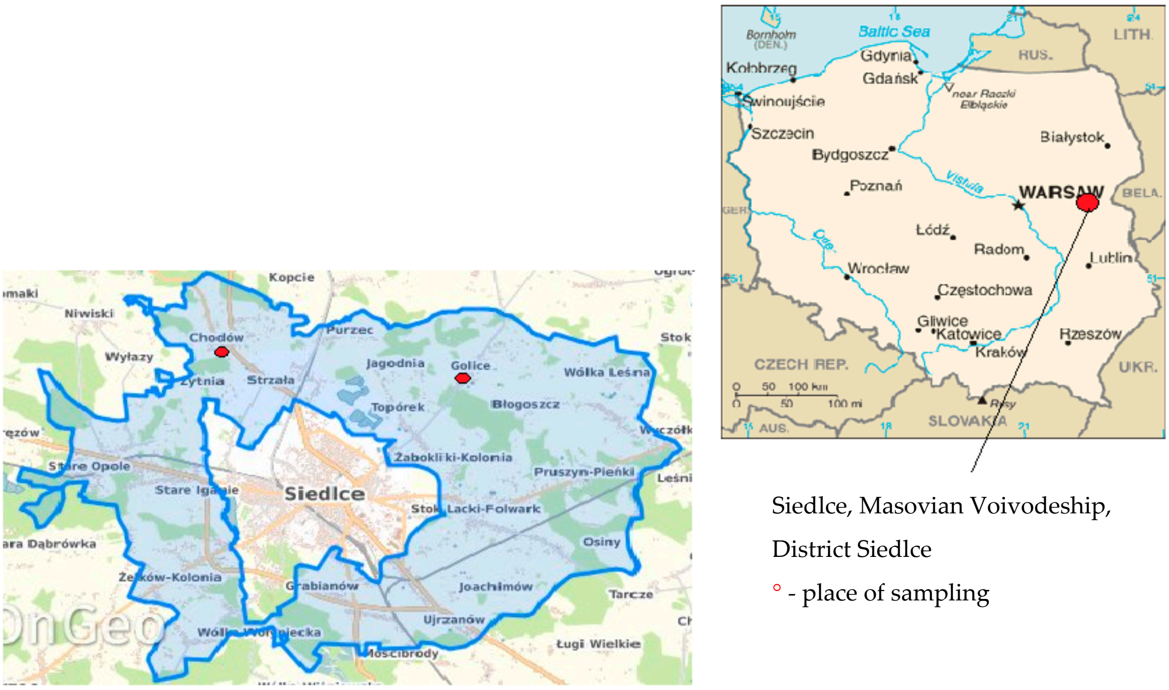

Figure S1. Location of Sampling for testing.

Table S1. Average values of temperature and precipitation in 2015–2017 (Siedlce district).

**Month**	**Years**					
	**2015**		**2016**		**2017**	
	**Temperature °C**	**Rainfall mm**	**Temperature °C**	**Rainfall mm**	**Temperature °C**	**Rainfall mm**
January	0.6	44.0	-4.5	10.9	-6.6	0.4
February	0.7	1.40	2.5	29.0	-1.3	15.9
March	4.6	12.0	3.5	33.5	5.5	25.1
April	8.6	35.2	9.1	28.7	6.9	59.6
May	13.6	38.6	15.1	54.8	13.9	49.5
June	17.7	19.2	18.4	36.9	17.8	57.9
July	20.1	49.0	19.1	35.2	16.9	23.6
August	22.1	7.5	18.0	31.7	18.4	54.7
September	15.0	23.4	14.9	13.6	13.9	80.1
October	7.0	22.8	7.0	69.8	9.0	53.0
November	4.7	18.7	2.4	19.5	4.1	21.3
December	3.6	8.2	0	11.3	2.7	15.9
The average annual	9.9	23.0	8.8	31.2	8.4	38.0

Data from the Zawady weather station.

Table S2. The pH value and the content of organic matter in the forest litter and soil samples (average value from the 0-20 cm layer) of the studied forest types.

Forest Type	Depth cm	Locality			
		Golice		Chodów	
		pH KCl	OS %	pH KCl	OS %
Coniferous forest	forest litter	4.51	78.3	4.01	90.5
	0-20	3.97	1.57	4.33	1.11
Deciduous forest	forest litter	5.19	67.8	4.99	58.0
	0-20	4.34	3.20	4.42	1.68
Mixed forest	forest litter	4.76	81.9	4.77	91.9
	0-20	4.15	2.42	4.27	3.39

OS - organic substance.
